# Laparoscopic functional fundoplication: a seven-step anti-reflux technique guided by Membrane anatomy landmarks

**DOI:** 10.1093/gastro/goaf094

**Published:** 2025-10-16

**Authors:** Yingru Li, Taicheng Zhou, Shuang Chen, Zhilong Yuan, Fuheng Liu, Wenchang Gan, Shaoyong Peng, Bing Zeng

**Affiliations:** Department of General Surgery, Hernia and Abdominal Wall Surgery, The Sixth Affiliated Hospital, Sun Yat-sen University, Guangzhou, Guangdong, P. R. China; Guangdong Provincial Key Laboratory of Colorectal and Pelvic Floor Diseases, The Sixth Affiliated Hospital, Sun Yat-sen University, Guangzhou, Guangdong, P. R. China; Biomedical Innovation Center, The Sixth Affiliated Hospital, Sun Yat-sen University, Guangzhou, Guangdong, P. R. China; Department of General Surgery, Hernia and Abdominal Wall Surgery, The Sixth Affiliated Hospital, Sun Yat-sen University, Guangzhou, Guangdong, P. R. China; Guangdong Provincial Key Laboratory of Colorectal and Pelvic Floor Diseases, The Sixth Affiliated Hospital, Sun Yat-sen University, Guangzhou, Guangdong, P. R. China; Biomedical Innovation Center, The Sixth Affiliated Hospital, Sun Yat-sen University, Guangzhou, Guangdong, P. R. China; Department of General Surgery, Hernia and Abdominal Wall Surgery, The Sixth Affiliated Hospital, Sun Yat-sen University, Guangzhou, Guangdong, P. R. China; Guangdong Provincial Key Laboratory of Colorectal and Pelvic Floor Diseases, The Sixth Affiliated Hospital, Sun Yat-sen University, Guangzhou, Guangdong, P. R. China; Biomedical Innovation Center, The Sixth Affiliated Hospital, Sun Yat-sen University, Guangzhou, Guangdong, P. R. China; Department of General Surgery, Hernia and Abdominal Wall Surgery, The Sixth Affiliated Hospital, Sun Yat-sen University, Guangzhou, Guangdong, P. R. China; Guangdong Provincial Key Laboratory of Colorectal and Pelvic Floor Diseases, The Sixth Affiliated Hospital, Sun Yat-sen University, Guangzhou, Guangdong, P. R. China; Biomedical Innovation Center, The Sixth Affiliated Hospital, Sun Yat-sen University, Guangzhou, Guangdong, P. R. China; Department of General Surgery, Hernia and Abdominal Wall Surgery, The Sixth Affiliated Hospital, Sun Yat-sen University, Guangzhou, Guangdong, P. R. China; Guangdong Provincial Key Laboratory of Colorectal and Pelvic Floor Diseases, The Sixth Affiliated Hospital, Sun Yat-sen University, Guangzhou, Guangdong, P. R. China; Biomedical Innovation Center, The Sixth Affiliated Hospital, Sun Yat-sen University, Guangzhou, Guangdong, P. R. China; Department of General Surgery, Hernia and Abdominal Wall Surgery, The Sixth Affiliated Hospital, Sun Yat-sen University, Guangzhou, Guangdong, P. R. China; Guangdong Provincial Key Laboratory of Colorectal and Pelvic Floor Diseases, The Sixth Affiliated Hospital, Sun Yat-sen University, Guangzhou, Guangdong, P. R. China; Biomedical Innovation Center, The Sixth Affiliated Hospital, Sun Yat-sen University, Guangzhou, Guangdong, P. R. China; Department of General Surgery, Hernia and Abdominal Wall Surgery, The Sixth Affiliated Hospital, Sun Yat-sen University, Guangzhou, Guangdong, P. R. China; Guangdong Provincial Key Laboratory of Colorectal and Pelvic Floor Diseases, The Sixth Affiliated Hospital, Sun Yat-sen University, Guangzhou, Guangdong, P. R. China; Biomedical Innovation Center, The Sixth Affiliated Hospital, Sun Yat-sen University, Guangzhou, Guangdong, P. R. China; Department of General Surgery, Hernia and Abdominal Wall Surgery, The Sixth Affiliated Hospital, Sun Yat-sen University, Guangzhou, Guangdong, P. R. China; Guangdong Provincial Key Laboratory of Colorectal and Pelvic Floor Diseases, The Sixth Affiliated Hospital, Sun Yat-sen University, Guangzhou, Guangdong, P. R. China; Biomedical Innovation Center, The Sixth Affiliated Hospital, Sun Yat-sen University, Guangzhou, Guangdong, P. R. China

## Introduction

The incidence of gastroesophageal reflux disease is on the rise in China, profoundly affecting patients’ quality of life. Although anti-reflux surgery effectively controls reflux, imprecise surgical techniques frequently result in a higher incidence of complications such as dysphagia [1]. Currently, membrane anatomy-based techniques have been widely used in various oncologic procedures to boost surgical precision [2], yet their application in anti-reflux surgery remains unreported. In this study, we described a seven-step technique guided by membranous anatomical landmarks and detailed how to precisely perform a functional fundoplication.

## Surgical procedure

We used the conventional five-hole method. The patient was placed in a reverse Trendelenburg position. The detailed surgical steps are as follows.

### Step 1: Dissection of the esophagus

**Figure 1.**
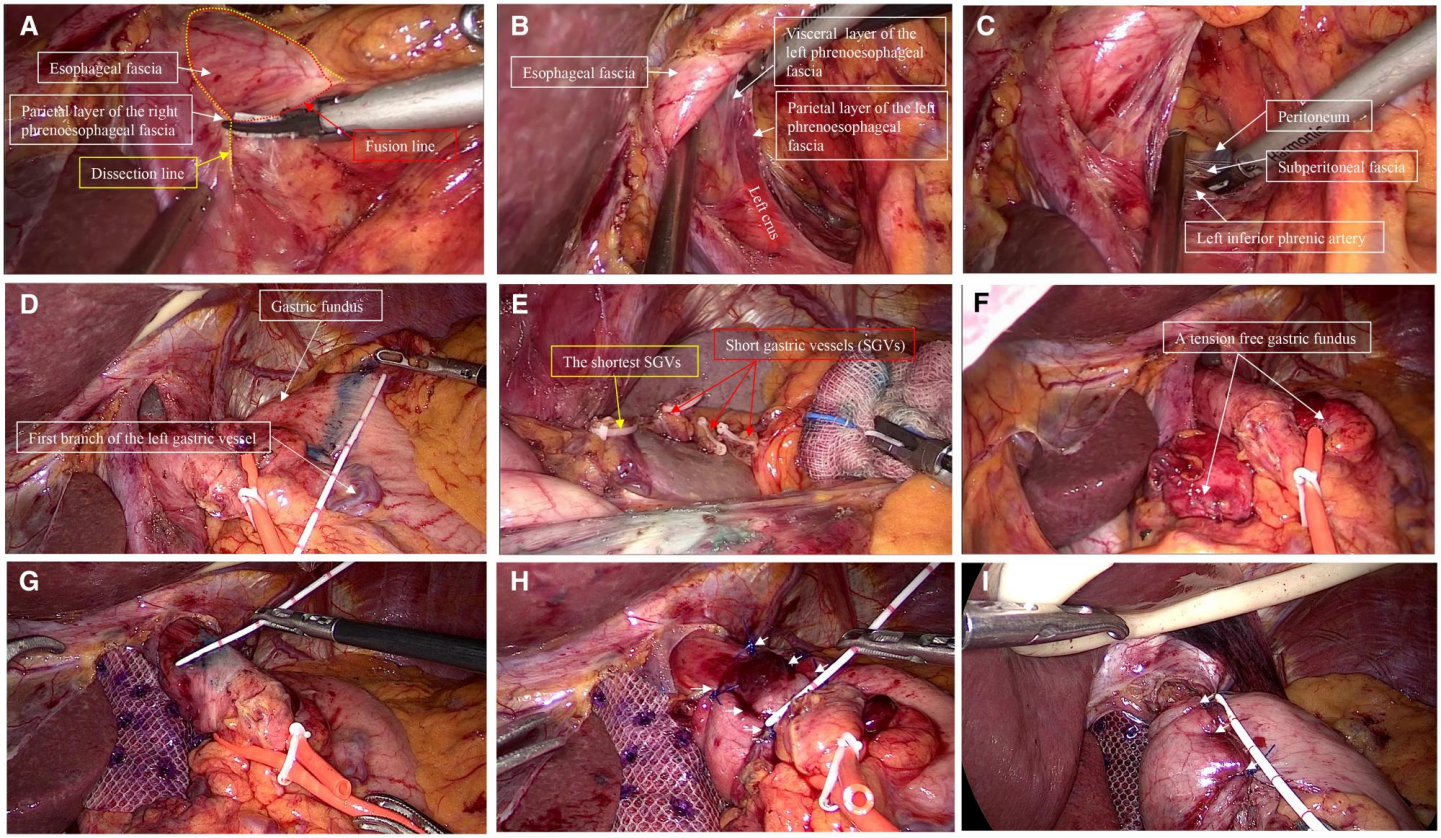
A seven-step anti-reflux technique guided by membranous anatomical landmarks. (A) The parietal layer of the right phrenoesophageal fascia. (B) The visceral and parietal layers of the left phrenoesophageal fascia. (C) The left inferior phrenic artery lay below the parietal layer of the left phrenoesophageal fascia. (D) The gastric fundus was marked using methylene blue. (E) A restrictive dissection method was used to divide the short gastric vessels. (F) A relatively free and adequate gastric fundus. (G) For Toupet fundoplication, a reverse marking method was used to mark the esophageal regions not to be wrapped. (H) Toupet fundoplication guided by the landmarks. (I) Nissen fundoplication guided by the landmarks.

First, the parietal layer of the right phrenoesophageal fascia (PEF) was dissected (Figure 1A). The visceral and parietal layers of the left PEF were then clearly identified (Figure 1B). Dissection was performed above the parietal layer of the left PEF (Figure 1C). Incising the transparent peritoneal ‘window’ facilitated access to the retro-gastroesophageal junction space (Supplementary Figure S1A and B). The esophagus was dissected from right to left to expand the surrounding space, revealing the anterior part of the PEF and the anterior vagal trunk (Supplementary Figure S1C), the parietal and visceral layers of the left PEF (Supplementary Figure S1D), the right PEF (also known as the infracardiac bursa) and the hiatal ligament of esophagus (Supplementary Figure S1E), and the posterior vagal trunk (Supplementary Figure S1F). Finally, a 3–4 cm non-stretched abdominal esophageal segment was obtained.

### Step 2: Restrictive dissection of the gastric fundus

For cases with tight type fundus (Supplementary Figure S2A), dissection is required for tension-free fundoplication. To avoid unnecessary dissection, the fundus area was first marked with methylene blue (Figure 1D). Dissection was typically performed from this marked line toward the splenic pole using the restrictive dissection method (Figure 1E). The posterior gastric vessels and their mesentery were divided as needed (Supplementary Figure S2B–D). Cases with gastric fundus branches (Supplementary Figure S2E) or esophageal branches (Supplementary Figure S2F) from the left inferior phrenic artery, the relevant vessels were also dissected when necessary. Adequate fundal mobility was confirmed by pulling the fundus to the right of the esophagus and releasing it—lack of retraction to the left indicated sufficient freedom (Figure 1F).

### Step 3: Crural reconstruction

The esophageal hiatus was closed by suturing the right and left crus with 2–0 non-absorbable sutures (Supplementary Figure S3A and B). The parietal layer of the PEF can be used for reconstruction. Notably, hiatus closure must be appropriately sized. In a non-tensioned state of the esophagus, the remaining gap of the esophageal hiatus should allow the esophagus to pass smoothly. A 1 cm gap is commonly considered appropriate (Supplementary Figure S3C). For cases with hiatal hernia, a patch can be used to reinforce the repair (Supplementary Figure S3D).

### Step 4: Selection of the area to be wrapped on the esophagus

It is crucial to determine the detailed location and extent of the esophagus to be encircled. For Toupet wrapping, measure the esophageal circumference in a non-tensioned state (Supplementary Figure S4A–C). Then, select the lower esophagus as the wrapping area. A reverse marking method was used to identify the regions not being wrapped, with a length of 2 cm (Supplementary Figure S4D and E) and a width of 1/4 of the circumference (Figure 1G). For Nissen wrapping, the suture points were marked with methylene blue (Supplementary Figure S4F). These enabled more accurate assessment of the degree and length of the wrapped flap.

### Step 5: Selection and identification of the fundus wrapping sites

To further assess the mobility of the gastric fundus, we used forceps to grasp the gastric fundus using a shoe-shine maneuver (Supplementary Figure S5A). Next, to identify the optimal wrap location and suture sites, the surgeon grasped the appropriate areas on both sides of the fundus and brought them closer to the esophagus, simulating the folded state (Supplementary Figure S5B). Finally, suture sites on the fundus were marked with methylene blue (Supplementary Figure S5C and D).

### Step 6: Fundoplication

For Toupet fundoplication, the flap was sutured first on the right, then on the left, ensuring even distribution of the three stitches (Figure 1H). For Nissen fundoplication, the middle stitch is placed first, followed by the upper and lower stitches (Figure 1I). Then, the flap valves were fixed to diaphragmatic crura (Supplementary Figure S6A–D). During the procedure, the esophagus should be kept without excessive tension, a condition referred to as ‘in-situ’ wrapping. This is to maintain esophageal compliance and allow the tension of the flap to achieve a functional state.

### Step 7: Tension evaluation

The tension of the flap can be evaluated by checking whether forceps could easily enter the gap between the flap and the esophagus on both sides (Supplementary Figure S7A–D). Moreover, we can assess if the flap is too tight by checking whether there are indentations on the encircled esophagus. Last, we can also evaluate if the esophagus is subjected to shearing forces from different directions by observing if it is bent at an angle.

## Discussion and conclusion

Despite the long-standing practice of anti-reflux surgery, previous studies still reported a high incidence of postoperative complications [3–6]. The reasons are multifaceted, including insufficient understanding of membranous anatomical structures, lack of meticulous surgical techniques, and unresolved technical details in the guidelines [7]. A typical example is whether the short gastric vessels should be divided [8, 9]. The SAGES guideline only discussed the two extremes of complete division or no division [7]. This posed significant confusion for surgeons. Here we present a detailed, step-by-step description of the anti-reflux surgery guided by the membrane anatomy landmarks.

This method has several advantages. First, the membrane anatomy-based dissection technique can facilitate reducing tissue damage and postoperative swelling. Second, the restrictive dissection method was used to selectively divided the short gastric vessels. It helps avoid unnecessary dissection and ensures a free gastric fundus without compromising its blood supply. Third, the landmark-guided approach helps the selection of an appropriate area for wrapping. More importantly, esophageal compliance and mechanical factors were also taken into account in the procedure.

In conclusion, this study helps systematize the procedures for anti-reflux surgery and training programs, and provide vital insights for future studies.

## Ethical approval

This study was approved by the ethical committee of the institution (2023ZSLYEC-618).

## Supplementary Material

goaf094_Supplementary_Data
