# Supplementary material for: Laparoscopic functional fundoplication: a seven-step anti-reflux technique guided by Membrane anatomy landmarks
Source: Gastroenterol Rep (Oxf). 2025 Oct 16;13:goaf094. doi: 10.1093/gastro/goaf094 (PMC12529097; doi:10.1093/gastro/goaf094)
Supplement: goaf094_Supplementary_Data [file goaf094_supplementary_data.zip › Supplementary Figure Legends final version.docx]

**Supplementary Figure Legends**

**Figure S1.** Landmarks in the dissection of the esophagus. (A and B) Transparent peritoneum and the gap behind the gastroesophageal junction; (C) The anterior part of the phrenoesophageal fascia and the anterior vagal trunk; (D) The parietal and visceral layers of the left phrenoesophageal fascia; (E) The visceral layer of the right phrenoesophageal fascia and hiatal ligament of esophagus; (F) The posterior vagal trunk.

**Figure S2.** Landmarks in the dissection of the gastric fundus. (A) Tight type gastric fundus and its attachment; (B–D) The posterior gastric vessels and their associated mesentery; (E) Gastric fundus branches; (F) Esophageal branches.

**Figure S3.** Crural reconstruction. (A) Continuous suture of the right and left crus posteriorly to the esophagus; (B) Three interrupted sutures with 2-0 non-absorbable suture to reinforce the reconstruction; (C) An appropriate space is left for the esophagus; (D) Reinforce the repair with patch when necessary.

**Figure S4.** Selection of the area to be wrapped on the esophagus. (A–C) Measure the circumference of the esophagus in a non-tensioned state; (D and E) Utilizing a reverse marking method to mark the regions that remain unwrapped; (F) Methylene blue was used to mark the suture points.

**Figure S5.** Selection and identification of the fundus wrapping sites. (A) Using a shoe-shine maneuver to assess the mobility of the gastric fundus; (B) Simulation of the folding state; (C and D) Marking the suture points in the gastric fundus.

**Figure S6.** Secure the flap valves. (A and B) The left and right flaps were sutured to the diaphragm respectively (Toupet); (C and D) The left and right flaps were sutured to the diaphragm respectively (Nissen).

**Figure S7.** Tension evaluation. (A and B) Forceps were inserted between the flap and the esophagus to assess the flap tension (Toupet); (C and D) Forceps were inserted between the flap and the esophagus to assess the flap tension (Nissen).
