# Supplementary figures and images for: Laparoscopic functional fundoplication: a seven-step anti-reflux technique guided by Membrane anatomy landmarks
Source: Gastroenterol Rep (Oxf). 2025 Oct 16;13:goaf094. doi: 10.1093/gastro/goaf094 (PMC12529097; doi:10.1093/gastro/goaf094)

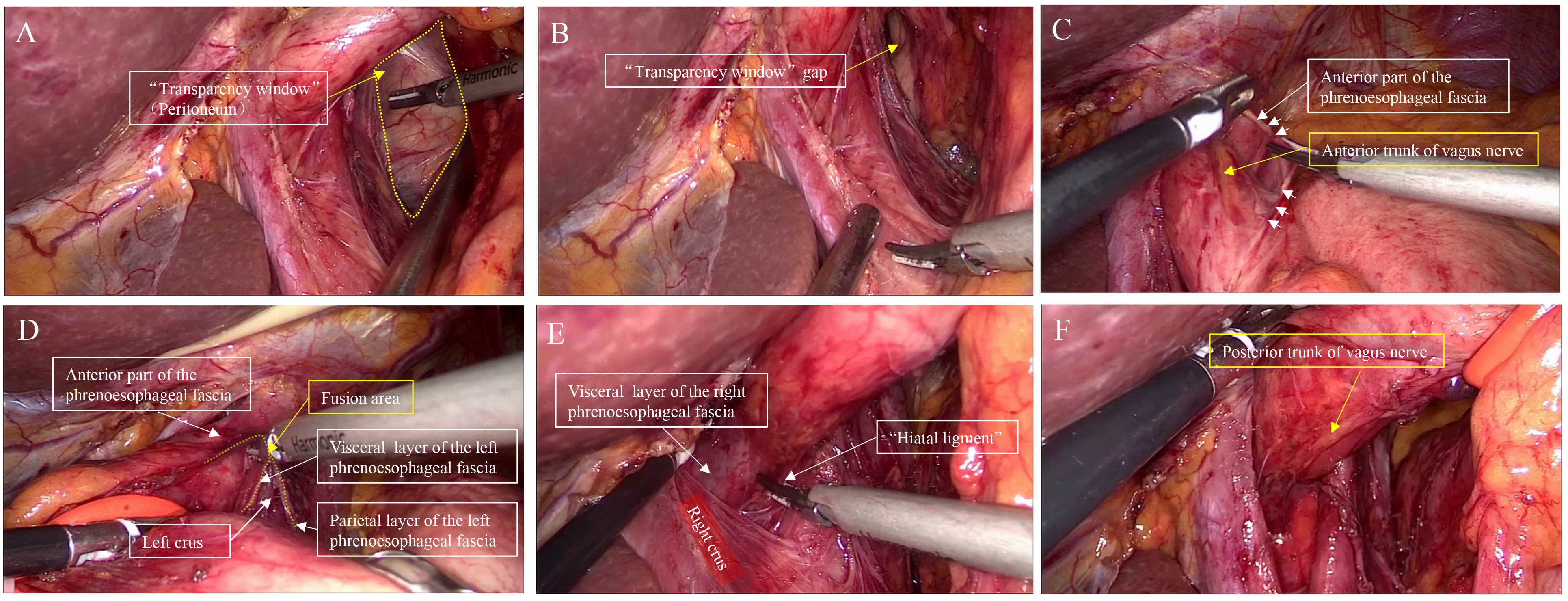

Supplement: goaf094_Supplementary_Data [file goaf094_supplementary_data.zip › Figure S1.tif]

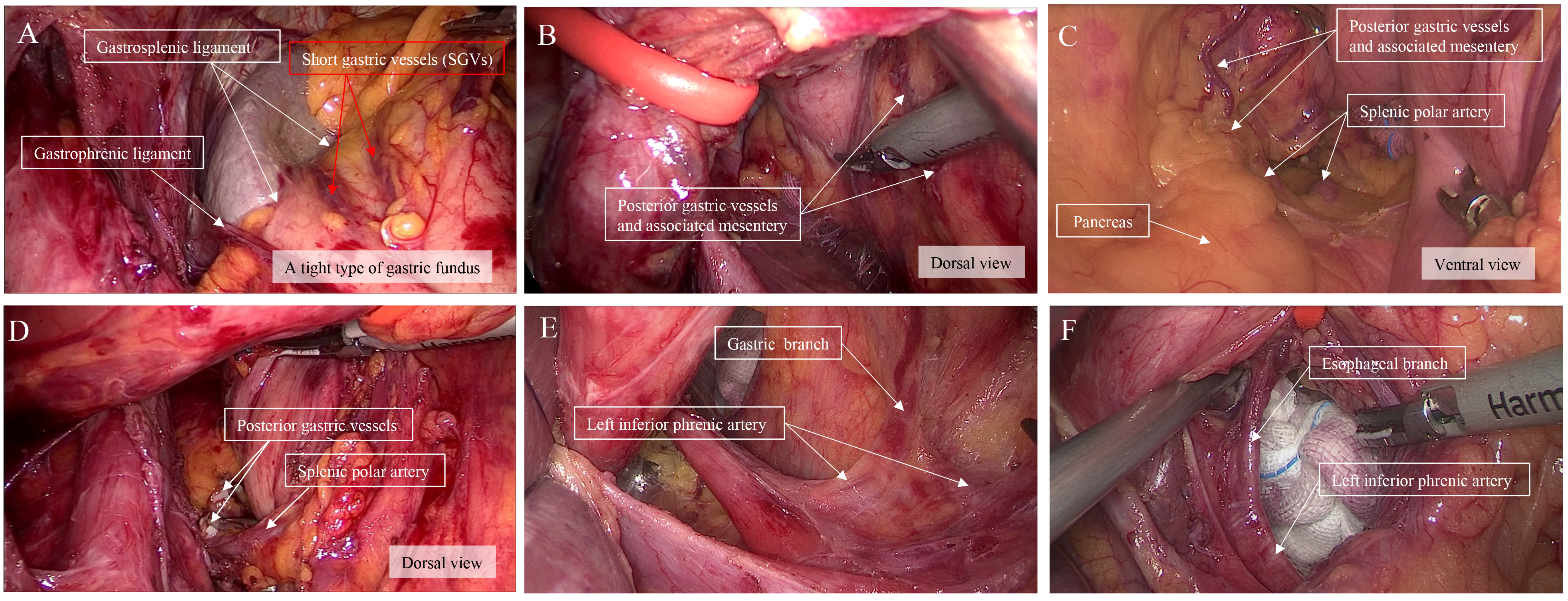

Supplement: goaf094_Supplementary_Data [file goaf094_supplementary_data.zip › Figure S2.tif]

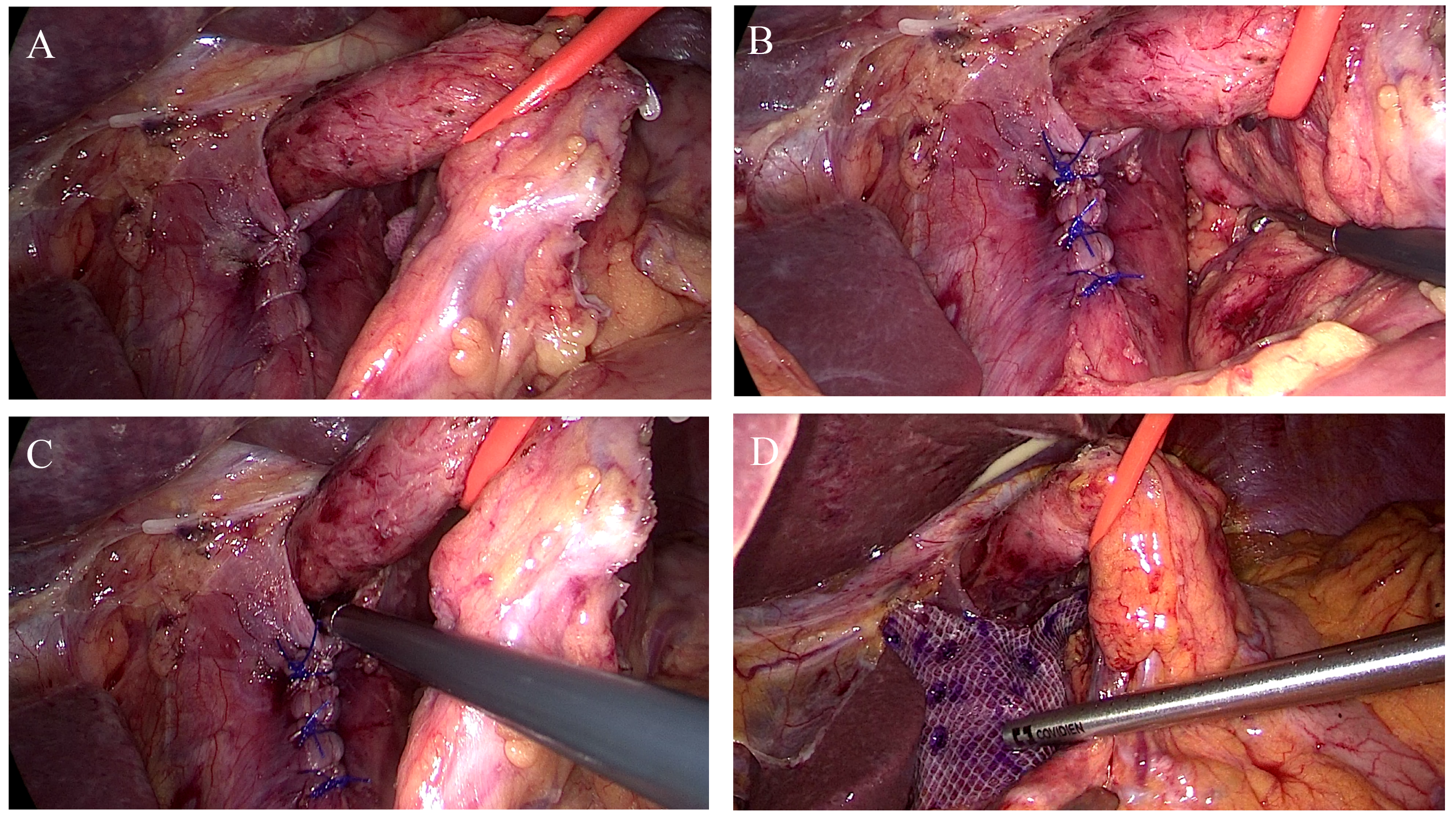

Supplement: goaf094_Supplementary_Data [file goaf094_supplementary_data.zip › Figure S3.tif]

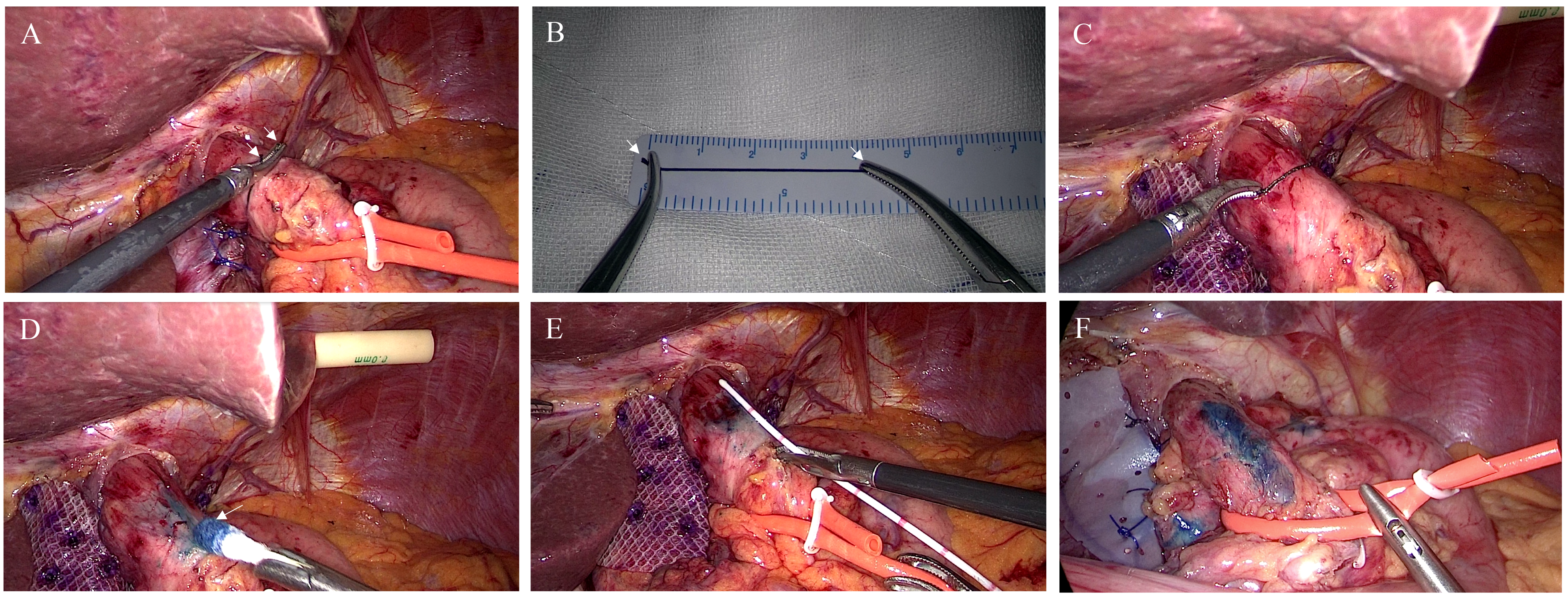

Supplement: goaf094_Supplementary_Data [file goaf094_supplementary_data.zip › Figure S4.tif]

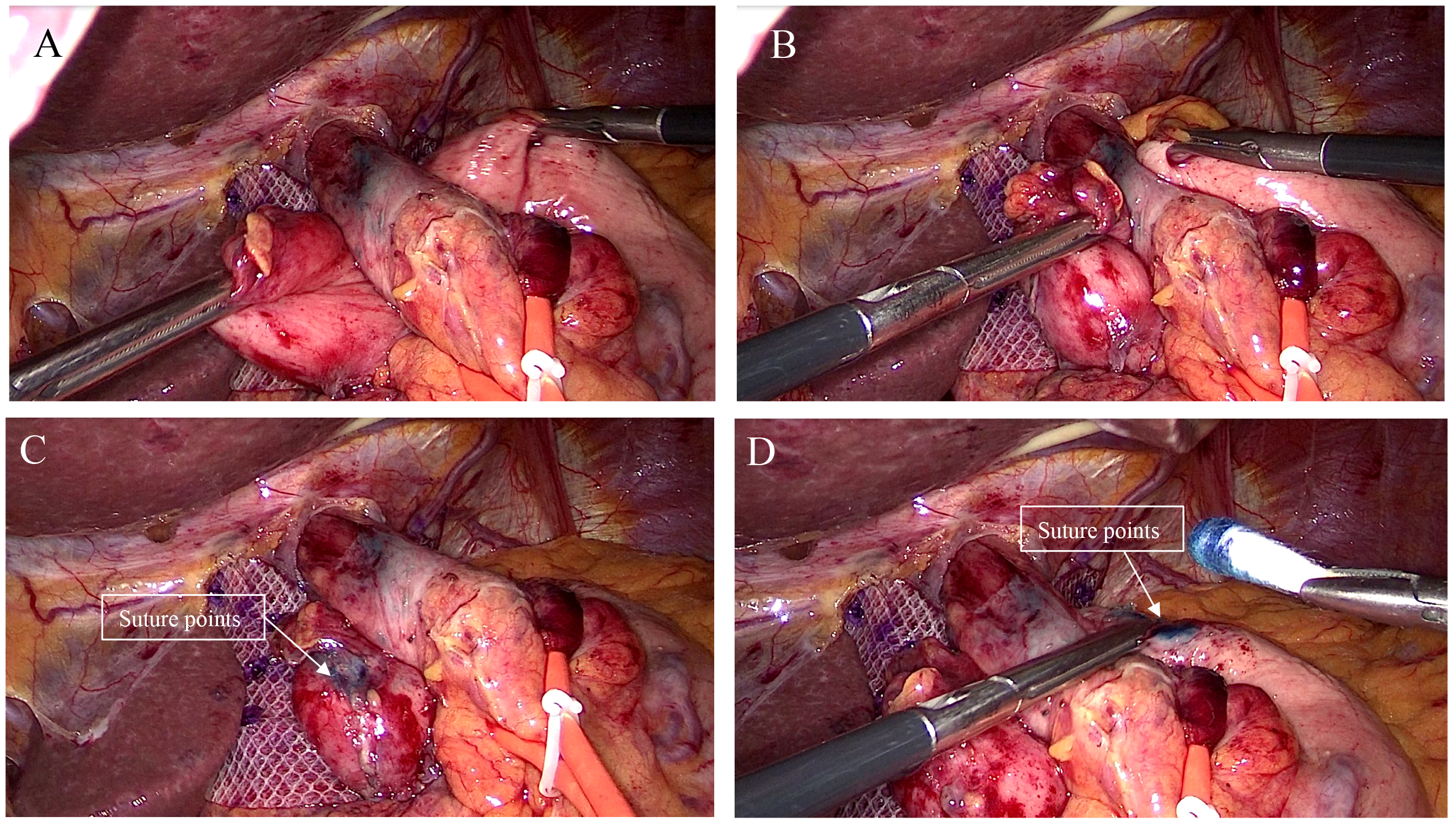

Supplement: goaf094_Supplementary_Data [file goaf094_supplementary_data.zip › Figure S5.tif]

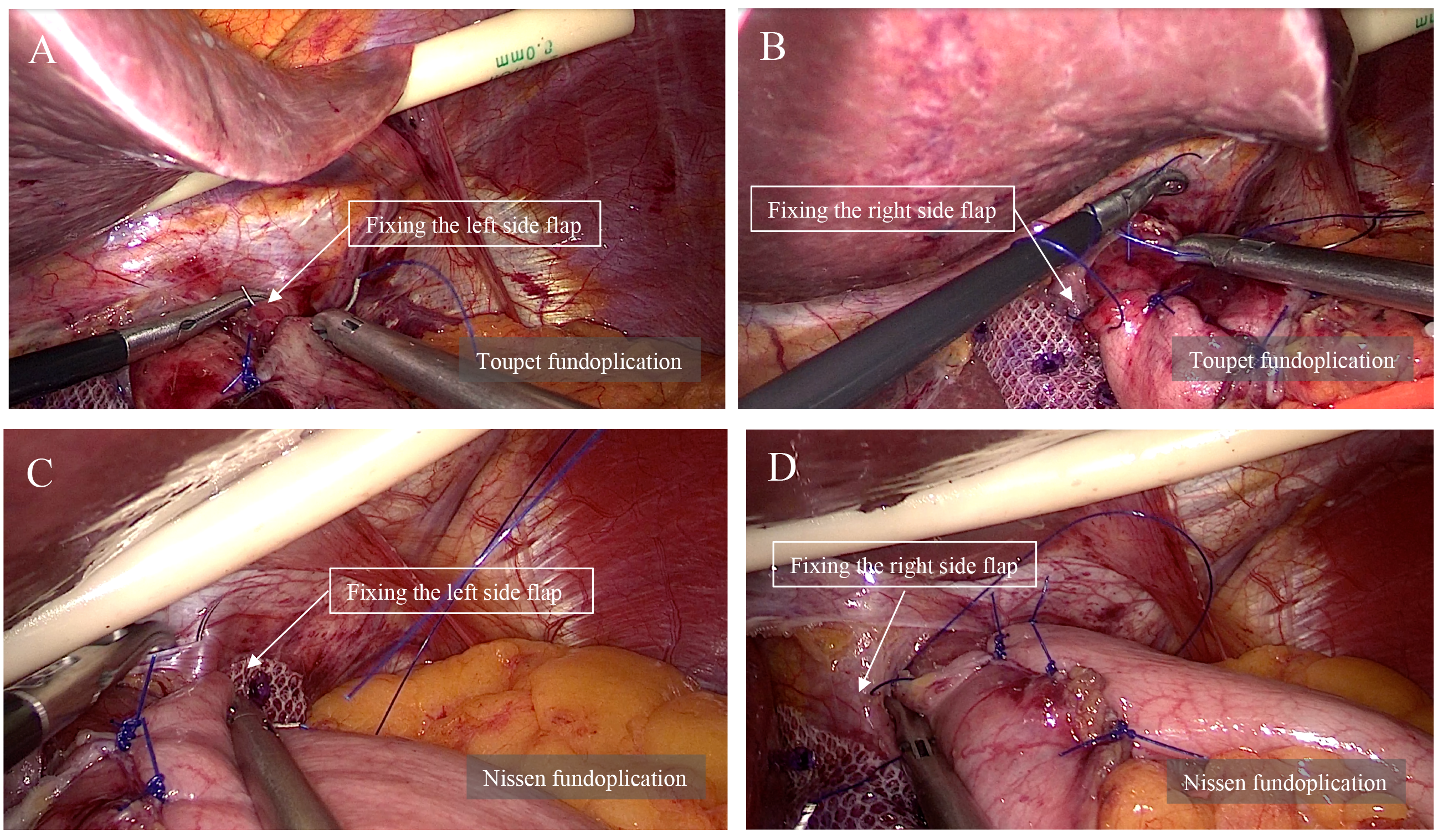

Supplement: goaf094_Supplementary_Data [file goaf094_supplementary_data.zip › Figure S6.tif]

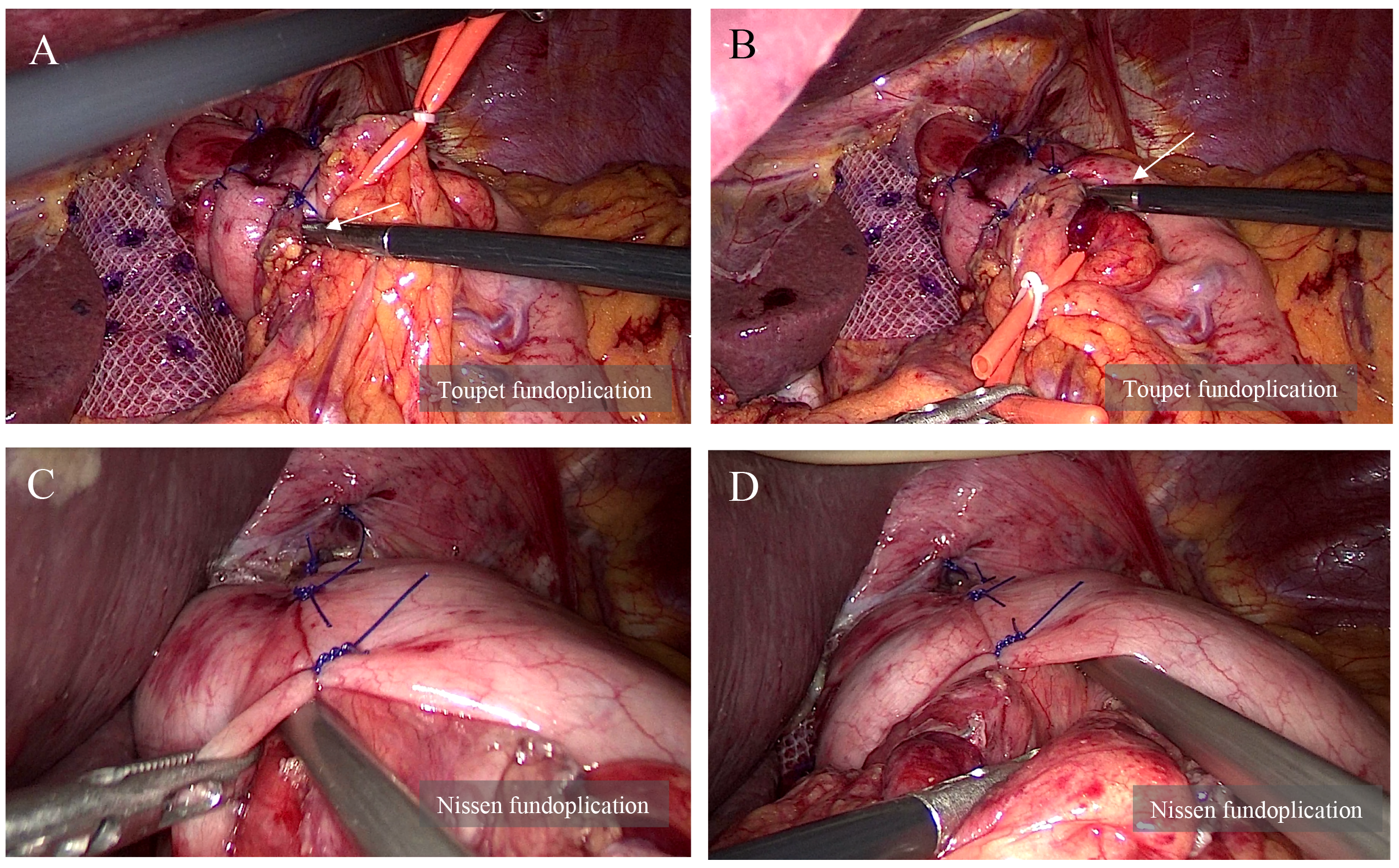

Supplement: goaf094_Supplementary_Data [file goaf094_supplementary_data.zip › Figure S7.tif]
